# Hypoalbuminemia and Risk of Portal Vein Thrombosis in Cirrhosis

**DOI:** 10.1016/j.gastha.2024.03.006

**Published:** 2024-03-13

**Authors:** Roberto Cangemi, Valeria Raparelli, Giovanni Talerico, Stefania Basili, Francesco Violi, PRO-LIVER Collaborators, Palasciano Giuseppe, D’Alitto Felicia, Palmieri Vincenzo Ostilio, Santovito Daniela, Di Michele Dario, Croce Giuseppe, Sacerdoti David, Brocco Silvia, Fasolato Silvano, Cecchetto Lara, Bombonato Giancarlo, Bertoni Michele, Restuccia Tea, Andreozzi Paola, Liguori Maria Livia, Perticone Francesco, Caroleo Benedetto, Perticone Maria, Staltari Orietta, Manfredini Roberto, De Giorgi Alfredo, Averna Maurizio, Giammanco Antonina, Granito Alessandro, Pettinari Irene, Marinelli Sara, Bolondi Luigi, Falsetti Lorenzo, Salvi Aldo, Durante-Mangoni Emanuele, Cesaro Flavio, Farinaro Vincenza, Ragone Enrico, Morana Ignazio, Andriulli Angelo, Ippolito Antonio, Iacobellis Angelo, Niro Grazia, Merla Antonio, Raimondo Giovanni, Maimone Sergio, Cacciola Irene, Varvara Doriana, Drenaggi Davide, Staffolani Silvia, Picardi Antonio, Vespasiani-Gentilucci Umberto, Galati Giovanni, Gallo Paolo, Davì Giovanni, Schiavone Cosima, Santilli Francesca, Tana Claudio, Licata Anna, Soresi Maurizio, Bianchi Giovanni Battista, Carderi Isabella, Pinto Antonio, Tuttolomondo Antonino, Ferrari Giovanni, Gresele Paolo, Fierro Tiziana, Morelli Olivia, Laffi Giacomo, Romanelli Roberto Giulio, Arena Umberto, Stasi Cristina, Gasbarrini Antonio, Gargovich Matteo, Zocco Maria Assunta, Riccardi Laura, Ainora Maria Elena, Capeci William, Martino Giuseppe Pio, Nobili Lorenzo, Cavallo Maurizio, Frugiuele Pierluigi, Greco Antonio, Pietrangelo Antonello, Ventura Paolo, Cuoghi Chiara, Marcacci Matteo, Serviddio Gaetano, Vendemiale Gianluigi, Villani Rosanna, Gargano Ruggiero, Vidili Gianpaolo, Di Cesare Valentina, Masala Maristella, Delitala Giuseppe, Invernizzi Pietro, Di Minno Giovanni, Tufano Antonella, Purrello Francesco, Privitera Graziella, Forgione Alessandra, Curigliano Valentina, Senzolo Marco, Rodríguez-Castro Kryssia Isabel, Giannelli Gianluigi, Serra Carla, Neri Sergio, Pignataro Pietro, Rizzetto Mario, Debernardi Venon Wilma, Svegliati Baroni Gianluca, Bergamaschi Gaetano, Masotti Michela, Costanzo Filippo, Corazza Gino Roberto, Caldwell Stephen Hugh, Angelico Francesco, Del Ben Maria, Napoleone Laura, Polimeni Licia, Proietti Marco, Raparelli Valeria, Romiti Giulio Francesco, Ruscio Eleonora, Severoni Andrea, Talerico Giovanni, Toriello Filippo, Vestri Annarita

**Affiliations:** 1 Department of Translational and Precision Medicine, Sapienza University of Rome, Rome, Italy; 2 Faculties of Nursing, Medicine and School of Public Health, University of Alberta, Edmonton, Alberta, Canada; 3 Internal Medicine, Azienda Ospedaliera San Giovanni Addolorata, Rome, Italy; 4 Department of Clinical Internal, Anesthesiological and Cardiovascular Sciences, Sapienza University of Rome, Rome, Italy; 5 Medicina Cardiocentro, Naples, Italy

**Keywords:** Albumin, Cirrhosis, Portal Vein Thrombosis

## Abstract

**Background and Aims:**

Hypoalbuminemia, as defined by serum albumin (SA) levels ≤35 g/L, is associated to venous and arterial thrombosis in general population and in patients at risk of cardiovascular disease. It is unknown if SA ≤35 g/L is also associated to portal vein thrombosis (PVT) in cirrhosis.

**Methods:**

Cirrhotic patients enrolled in the Portal vein thrombosis Relevance On Liver cirrhosis: Italian Venous thrombotic Events Registry (PRO-LIVER) study (n = 753), were followed-up for 2 years to assess the risk of PVT, that was diagnosed by Doppler ultrasonography. Child-Pugh classes, Model for End-Stage Liver Disease score, presence of hepatocellular carcinoma and laboratory variables including SA, D-dimer, and high-sensitivity C-reactive protein (hs-CRP) were measured at baseline.

**Results:**

SA ≤35 g/L was detected in 52% of patients. A logistic multivariate regression analysis showed that higher Child-Pugh class, hepatocellular carcinoma and thrombocytopenia were significantly associated to SA ≤35 g/L. In a subgroup of patients where data regarding hs-CRP and D-dimer were available, SA ≤35 g/L was inversely associated with hs-CRP and D-dimer. During the follow-up, a total of 61 patients experienced PVT. A Kaplan Meier survival analysis showed SA ≤35 g/L was associated to increased risk of PVT compared to SA >35 g/L (*P* = .005). A multivariate Cox proportional hazards regression analysis showed that male sex, lower platelet count, and SA ≤35 g/L remained associated to PVT after adjusting for confounding factors.

**Conclusion:**

Cirrhotic patients with SA ≤35 g/L are at higher risk of experiencing PVT compared to those with SA >35 g/L and could be considered as potential candidates to anticoagulant prophylaxis for PVT prevention.

## Introduction

For decades bleeding has been considered the hallmark of liver cirrhosis as patients presented changes of clotting system activation such as prolongation of prothrombin time and activated partial thromboplastin test as well as thrombocytopenia; this triad has been suggested to cause a coagulopathy so predisposing to bleeding.^1^ By time, evidence has been accumulated that in cirrhosis bleeding is rarely systemic and occurs prevalently in the gastrointestinal tube for hemodynamic reasons more than for changes of clotting system.^2^ Also, deeper analysis of clotting system demonstrated that the in vitro changes of clotting system did not reflect the activation of clotting system in vivo that, conversely, appeared to be overactivated.^3^^,^^4^ Thus, our groups was among the first to show that cirrhotic patients have an enhanced generation of thrombin, that is more evident in patients with advanced liver disease and is associated with systemic fibrinolysis.^4^ Also, we found that clotting activation was more marked in the portal circulation coincidentally with a low-grade endotoxemia suggesting a role for gut dysbiosis in translocating gut endotoxins such as lipopolysaccharides into portal and systemic circulation with ensuing lipopolysaccharides-mediated clotting activation.^5^ Other factors that may elicit clotting activation in cirrhosis include an imbalance between clotting factors and anticoagulant biosynthesis with a more profound reduction of anticoagulants compared to clotting factors.^6^ The prothrombotic state of cirrhosis is of clinical interest as it helps explaining its association with portal vein thrombosis (PVT), whose prevalence ranges from 0.6% to 16% by angiography or surgical series and from 10% to 25% by ultrasonography series.^2^ PVT is a life-threatening disease as it can precipitate vascular events, such as gastrointestinal bleeding and mesenteric thrombosis, secondary to mesenteric vein thrombosis extension and seriously limits performing liver transplantation.^7^ The incidence of PVT has been investigated by The Portal vein thrombosis Relevance On Liver cirrhosis: Italian Venous thrombotic Events Registry (PRO-LIVER) study including 753 Child-Pugh A, B, C classes of cirrhotic patients, that examined prevalence and incidence of PVT during a follow-up of 2 years; PVT prevalence was 17% while the incidence of PVT was 4.1 per 100 patient-years in PVT-free patients and 18.9 per 100 patient-years in those with PVT at admission.^8^^,^^9^ Identification of PVT predictors is, therefore, relevant to counteract PVT and its sequelae.

Albumin is synthetized by liver cells and is the most important circulating protein, that exerts multiple functions beyond its role in regulating colloid osmotic pressure.^10^ Thus, albumin transports proteins, fatty acids or drugs and exerts an anti-inflammatory activity via its thiol groups that allow to quench reactive oxidant species (ROS) so modulating the redox status.^10^^,^^11^ More interestingly, albumin encompasses anticoagulant properties via a heparin-like activity and inhibition of platelet aggregation.^10^ Retrospective studies reported an inverse relationship of serum albumin (SA) with previous PVT and incident PVT^12^, ^13^, ^14^, ^15^ while we did not find such association in the prospective analysis of the PRO-LIVER study^9^; however specific SA cut-off to identify patients at risk of PVT was not investigated. This issue is clinically relevant as hypoalbuminemia, as defined by SA ≤35 g/L^10^ has been discovered as cut-off to identify patients at risk of venous and arterial thrombosis in a study including > 100.000 without prior cardiovascular disease and in a meta-analysis including patients at risk of cardiovascular disease suggesting that hypoalbuminemia is a marker of ongoing prothrombotic state.^16^ To investigate if hypoalbuminemia is also independently associated with incident PVT, we analyzed the impact of SA ≤35 g/L on incident PVT in the PRO-LIVER study.

## Methods

### Study Design

The PRO-LIVER study is an Italy-based, prospective multicenter study with the primary objective of determining the prevalence of PVT and its incidence in a cohort of patients with cirrhosis of various etiologies and severity, during a 2-year follow-up period.

A detailed description of the entire study design has been previously documented in separate publications.^8^^,^^9^

The Italian Society of Internal Medicine co-ordinates all regional centers (refer to Online Appendix) participating in the study. These centers adhered to the same standard of care and formed a network for recruiting and monitoring cirrhotic patients. The study was conducted in compliance with the European Union Note for Guidance on Good Clinical Practice and the Declaration of Helsinki. Informed consent was obtained from all participants enrolled in the study. The initiation of the study followed local and ethical approval requirements (ClinicalTrials.gov Identifier: NCT01470547). Center participation in the registry was voluntary and not financially sponsored.

### Study Population

All consecutive cirrhotic patients referred to the 43 participating centers were included in the study. The sole exclusion criterion was the presence of concurrent extra-hepatic neoplasms. Consequently, this study encompassed patients with cirrhosis, irrespective of its etiology or severity, even in cases complicated by hepatocellular carcinoma (HCC).

At the baseline, medical histories, anthropometric data, and the severity of cirrhosis were recorded. The Child–Pugh score^17^ and the Model for End-Stage Liver Disease (MELD) score^18^ were assessed to establish the severity of liver disease. Among the laboratory variables, prothrombin time, total bilirubin, SA, and serum creatinine were mandatory for the computation of the Child–Pugh and MELD scores. However, investigators had the discretion to include additional laboratory parameters in the standard form as deemed necessary.

### PVT Evaluation and Definition

A Doppler ultrasound examination of the portal vein's main trunk, its branches, and tributaries was a requisite procedure to assess the presence of PVT.

According to predefined study criteria, the suspicion of PVT was initially raised upon the detection of solid endoluminal material in the main trunk of the portal vein and/or its branches. Confirmation of PVT was subsequently obtained through the observation of a filling defect during Doppler examination.

### Data Collection and Validation

In each center, data were collected using an electronic case report form. Data were transferred to the web-central database (Co-ordination Center: Sapienza-University of Rome). Using a validation plan integrated in the data entry software, data were checked for missing or contradictory entries and values out of the normal range. A final database was created and validated by the study co-ordinators.^8^

Patient identification names were recorded at the individual participating centers but were not transferred to the central database. Instead, patients were identified using unique serial numbers assigned to each center.

### Statistical Analysis

Categorical variables are reported as counts and percentages and continuous variables as mean ± standard deviation, or medians and interquartile ranges (IQRs). Differences between percentages were assessed by chi-square or Fisher exact tests. All continuous variables were tested for normality with the Shapiro-Wilk test. Student unpaired t-tests were used for normally distributed continuous variables. Mann-Whitney U test was used for not-normally distributed continuous variables. The bivariate and multivariate effects of prognostic factors on thrombotic events were assessed by means of logistic regression models. Wald confidence intervals (CIs) and tests for odds ratios and adjusted odds ratio were computed based on the estimated standard errors. Survival curves were estimated using the Kaplan-Meier product-limit estimator and compared using the log-rank test. Cox proportional hazards analysis was used to calculate the adjusted hazard ratios (HRs) and 95% CI for each clinical variable. Only *P* values < .05 were considered statistically significant. All tests were 2-tailed, and analyses were performed using computer software packages (IBM SPSS Statistics 27).

## Results

The clinical and laboratory features of the cohort, which comprised patients suffering from cirrhosis and were part of the Pro-Liver study, have previously been documented.^8^

Briefly, 753 consecutive patients (513 males, 241 females), with an average age of 64.4 ± 12.2 years, all of whom had liver cirrhosis, were recruited from 43 participating medical centers, and were included in the current analysis (Table 1). Notably, the predominant etiologies for cirrhosis in this cohort were viral and alcoholic.

**Table 1:** Baseline Clinical Characteristics of Patients With Albumin Levels Above or Below the Median Values (35 g/L)

Variables	All patients (N = 753)	Patients with albumin ≤35 g/L (N = 391)	Patients with albumin >35 g/L (N = 362)	*P*
Age, y	64.4 ± 12.2	64.8 ± 12.0	63.9 ± 12.3	.282
Male sex, N (%)	513 (68)	273 (70)	240 (66)	.300
Etiology, N (%)				<.001
ALD, N (%)	186 (25)	122 (31)	64 (18)	
Viral, N (%)	332 (44)	160 (41)	172 (47)	
Autoimmune, N (%)	18 (2)	7(2)	11 (3)	
NASH, N (%)	43 (6)	17 (4)	26 (7)	
Mixed, N (%)	102 (14)	48 (12)	54 (15)	
Others/unknown, N (%)	72 (10)	37 (10)	35 (10)	
Child–Pugh class				<.001
Class a, N (%)	395 (53)	101 (26)	294 (81)	
Class B, N (%)	260 (35)	198 (51)	62 (17)	
Class C, N (%)	98 (13)	92 (24)	6 (2)	
MELD score	10 [8–14]	12 [9–15]	8 [7–11]	<.001
HCC, N (%)	154 (20)	102 (26)	52 (14)	<.001
Bilirubin, (mg/dL)	1.2 [0.8–2.24]	1.7 [1.0–2.9]	1.0 [0.7–1.4]	<.001
PT-INR	1.3	1.37 ± 0.33	1.21 ± 0.31	<.001
Serum creatinine (mg/dL)	0.8 [0.7–1.0]	0.8 [0.7–1.0]	0.8 [0.7–1.0]	.321
Platelet count (×10^3^/ΜL)	98 [68–138]	89 [63–127]	110 [76–156]	<.001
Ascites				<.001
Absent, N (%)	464 (61)	166 (42)	298 (82)	
Responsive to diuretic therapy, N (%)	217 (29)	158 (40)	59 (16)	
Refractory, N (%)	72 (10)	67 (17)	5 (1)	
Encephalopathy				<.001
Absent, N (%)	636 (85)	295 (75)	341 (94)	
Mild, N (%)	104 (14)	85 (22)	19 (5)	
Moderate to severe, N (%)	13 (2)	11 (3)	2 (1)	
Anticoagulants, N (%)	98 (13)	52 (13)	46 (13)	.809
Warfarin, N (%)	26 (3)	8 (2)	18 (5)	.063
LMWH, N (%)	60 (8)	36 (9)	24 (7)	
Fondaparinux, N (%)	12 (2)	8 (2)	4 (1)	

Data are expressed as mean ± standard deviation, or median [interquartile range] or number (percentage).

ALD, alcohol-associated liver disease; HCC, Hepatocellular carcinoma; LMWH, low-molecular-weight heparin; MELD, Model for End-Stage Liver Disease; NASH, nonalcoholic steatohepatitis; PT-INR, prothrombin time-international normalized ratio.

Roughly half of the patients exhibited a moderate to severe disease severity, as assessed by the Child-Pugh score (35% in class B and 13% in class C). HCC was present at the time of admission in 20% of cases. A significant proportion of the participants had no signs of ascites (61%) or encephalopathy (85%). Thirteen percent of participants was treated with anticoagulants (warfarin, low-molecular-weight heparins, or fondaparinux).

At baseline, the median and IQR values for SA were 35 [30–39] g/L. Baseline clinical characteristics of patients, categorized according to SA levels (≤35 g/L for individuals with hypoalbuminemia and >35 g/L for those with albumin levels within the normal range) are reported in Table 1. Notably, as 35 g/L coincided with the median SA value in our cohort, approximately half of the patients (52%) exhibited SA levels ≤35 g/L. Patients with albumin levels ≤35 g/L were more likely to have an alcoholic etiology, a higher Child-Pugh class, and MELD score, a greater incidence of HCC (26% vs 14%, *P* < .001), an increased likelihood of having ascites, experiencing encephalopathy, and exhibited higher levels of bilirubin and lower platelet count; conversely, there were no significant differences observed of sex, age, or creatinine levels between the 2 groups.

### Variables Associated With Low Albumin Levels

A logistic multivariate regression analysis showed that clinical factors independently associated to SA levels ≤ 35g/L included a higher Child-Pugh class and HCC. When laboratory parameters were incorporated into the multivariate model, Child-Pugh classes, HCC, and lower platelet count remained significantly associated with SA ≤35 g/L, after adjustment for potential confounding variables (Table 2).

**Table 2:** Logistic Regression Analyses: Clinical Variables Independently Associated With Albumin Values ≤ 35 g/L

Model A	OR:	95% CI	*P*
Child classes	7.834	5.641 (10.879)	<.001
HCC	2.120	1.349 (3.332)	.001

Model B	OR:	95% CI	*P*
Child classes	9.415	6.408 (13.832)	<.001
HCC	2.466	1.477 (4.119)	.001
PLT count (×10^3^/μL)	0.996	0.993 (0.999)	.011

Model A: adjusted for cirrhosis etiology, MELD, score, presence of ascites and encephalopathy.

Model B: adjusted for variables already included in Model A, bilirubin, and INR.

CI, confidence interval; HCC, Hepatocellular carcinoma; OR, odd ratio; PLT, platelets.

### Relationship Between Albumin Levels, Inflammatory Biomarkers, and D-Dimer

Data on high-sensitivity C-reactive protein (hs-CRP) were available for 264 patients out of the 753 patients (the demographic and clinical characteristics of this specific patient subgroup are detailed in Table A1). In this subset, individuals with SA ≤35 g/L showed higher hs-CRP levels compared to those with SA >35 g/L (2.9 [0.9–10.3] vs 1 [0.28–3.0] mg/L; *P* < .001). Furthermore, data on D-dimer levels were available for 112 patients (the demographic and clinical characteristics of this specific patient subgroup are detailed in Table A2). Patients with albumin levels ≤35 g/L exhibited higher D-dimer levels compared to those with SA >35 g/L (1210 [451–2783] vs 310 [135–646] ng/ml; *P* < .001).

### Follow-Up: Long Term PVT Events

Seven hundred and fifty-three patients diagnosed with cirrhosis were subjected to a follow-up period with a median duration of 21 months, and an IQR of 6.7–24 months. This resulted in the accrual of 1008 patient-years of observational data. Throughout the follow-up timeframe, a total of 61 patients, constituting 8% of the cohort, experienced PVT. Patients with PVT were more prevalently male, had lower baseline platelet counts, and demonstrated a proclivity towards a greater incidence of mild encephalopathy (Table 3). Conversely, no discernible disparities in age, Child-Pugh score, MELD-score, cirrhosis etiology, HCC occurrence, or ascites incidence at the time of hospital admission were observed between the 2 groups. A Kaplan Meier survival analysis showed an increased risk of PVT for patients with baseline SA values ≤ 35 g/L (*P* = .005), compared to patients with albumin values > 35 g/L (Figure).

**Table 3:** Clinical and Laboratory Characteristics in Cirrhotic Patients Experienced Portal Vein Thrombosis (PVT) During the Follow-Up

Variables	Patients without PVT (N = 692)	Patients with incident PVT (N = 61)	*P*
Age, y	64.8 ± 12.6	64.2 ± 14.6	.735
Male sex, N (%)	463 (67)	50 (82)	.016
Etiology, N (%)			.898
ALD, N (%)	170 (25)	16 (26)	
Viral, N (%)	308 (44)	24 (40)	
Autoimmune, N (%)	17 (2)	1 (2)	
NASH, N (%)	40 (6)	3 (5)	
Mixed, N (%)	91 (13)	11 (18)	
Others/unknown, N (%)	66 (9)	6 (10)	
Child–Pugh class			.901
Class A, N (%)	367 (53)	32 (52)	
Class B, N (%)	234 (34)	22 (36)	
Class C, N (%)	91 (13)	11 (7)	
MELD score	11 [8–16]	11 [8–12]	.156
HCC, N (%)	139 (20)	13 (21)	.819
Albumin, (g/L)	3.4 ± 0.6	3.4 ± 0.6	.998
Bilirubin, (mg/dL)	1.3 [0.8–2.9]	1.3 [0.9–1.8]	.248
PT-INR	1.30 ± 0.34	1.31 ± 0.20	.816
Serum creatinine (mg/dL)	0.8 [0.7–1.0]	0.8 [0.7–1.0]	.713
Platelet count (×10^3^/μL)	100 [71–144]	72 [52–108]	<.001
Ascites			.592
Absent, N (%)	429 (62)	35 (57)	
Responsive to diuretic therapy, N (%)	196 (28)	21 (34)	
Refractory, N (%)	67 (10)	5 (8.2)	
Encephalopathy			.097
Absent, N (%)	590 (85)	46 (75)	
Mild, N (%)	90 (13)	14 (23)	
Moderate to severe, N (%)	12 (2)	1 (2)	
Baseline use of anticoagulants, N (%)	91 (13)	7 (11)	.709
Warfarin, N (%)	25 (4)	1 (2)	.884
LMWH, N (%)	55 (8)	5 (8)	
Fondaparinux, N (%)	11 (2)	1 (2)	

Data are expressed as mean ± standard deviation, or median [interquartile range] or number (percentage).

ALD, alcohol-associated liver disease; HCC, Hepatocellular carcinoma; MELD, Model for End-Stage Liver Disease; NASH, nonalcoholic steatohepatitis; PT-INR, prothrombin time-international normalized ratio.

**Figure:**
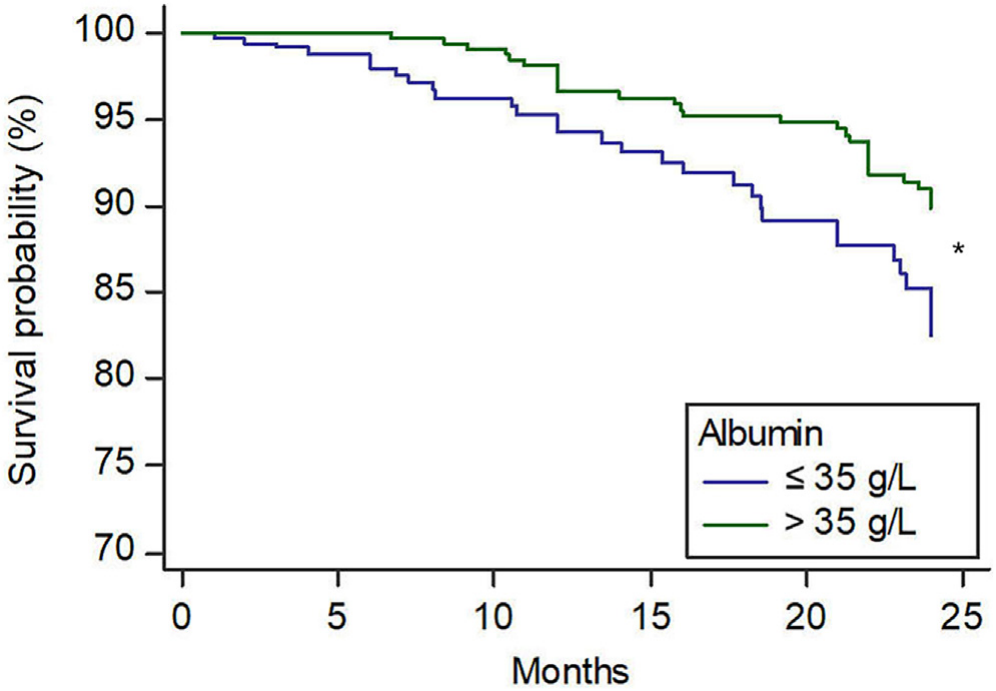
Kaplan–Meier estimates of time to PVT according to baseline albumin levels during the long-term follow-up.

Univariate COX regression analyses showed that male sex, Child-Pugh class, baseline SA levels, and platelet count were associated to PVT during the follow-up (Table 4). A multivariate COX regression analysis showed that male sex, lower platelet count, and SA ≤35 g/L remained associated to PVT during the follow-up, after adjusting for confounding factors (Table 5).

**Table 4:** Variables Associated to PVT During the Follow-Up: Univariate COX Regression Analyses

Variables	HR	95% CI	*P*
Age, y	1.000	0.980 (1.020)	.990
Male sex, N (%)	2.269	1.181 (4.360)	.016
Etiology			
ALD	1.092	0.601 (1.982)	.773
Viral	0.899	0.688 (1.175)	.437
Autoimmune	0.871	0.442 (1.717)	.691
NASH	0.958	0.709 (1.295)	.781
Mixed	1.078	0.939 (1.237)	.288
Others/unknown	1.006	0.869 (1.165)	.939
Child–Pugh class	1.468	1.036 (2.081)	.031
MELD score	0.988	0.975 (1.001)	.068
HCC, N (%)	1.497	0.810 (2.766)	.198
Albumin (g/L)	0.612	0.399 (0.938)	.024
Albumin ≤35 g/L	2.017	1.217 (3.343)	.006
Bilirubin (mg/dL)	0.990	0.977 (1.002)	.111
PT-INR	0.996	0.987 (1.004)	.331
Serum creatinine (mg/dL)	0.995	0.987 (1.004)	.277
PLT count (×10^3^/ΜL)	0.987	0.981 (0.993)	<.001
Baseline use of anticoagulants	0.856	0.378 (1.939)	.710

ALD, alcohol-associated liver disease; CI, confidence interval; HCC, Hepatocellular carcinoma; HR, hazard ratio; NASH, Nonalcoholic steatohepatitis; PLT, platelets; PT-INR, prothrombin time-international normalized ratio.

**Table 5:** COX Regression Analysis

Variables	HR	95% CI	*P*
Male sex	2.009	1.044 (3.866)	.037
Platelet count (×10^3^/ΜL)	0.988	0.982 (0.994)	<.001
Albumin ≤35 g/L	1.679	1.009 (2.795)	.046

Variables independently associated to PVT during the follow-up.

After adjusting for Child–Pugh classes and MELD, score.

CI, confidence interval; HR, hazard ratio.

### Long Term PVT Events in Patients Free from PVT at Baseline

In our cohort, PVT incidence was more common in patients who already showed a PVT at baseline (20% PVT recurrence among patients with PVT at baseline vs 6% new PVT among PVT-free patients, *P* < .001). Thus, the relationship between albumin and PVT incidence was reanalyzed, after excluding 126 patients with PVT at baseline.

In remaining 627 patients, 36 new PVT were observed during the follow-up time (21 among the 307 patients with SA ≤35 g/L vs15 among the 320 patients with SA >35 g/L. A COX regression analysis confirmed that SA ≤35 g was associated with incident PVT during the follow-up (HR: 2.233; 95% CI: 1.149–4.341; *P* = .018), after adjusting for confounding factors.

### Long Term PVT Events in Patients Free from HCC at Baseline

As an increased risk of PVT has been reported in HCC patients,^19^ the relationship between albumin and PVT was reanalyzed, after excluding 155 patients with HCC.

Patients without baseline HCC were younger (63 ± 12.3 vs 69 ± 10.5 years; *P* < .001) and less likely to be male (64 vs 83%; *P* < .001) compared to patient with HCC. In this cohort of 599 patients (386 men and 213 women), 47 new cases of PVT were observed during the follow-up, with 29 out of 289 (10%) occurring in patients with SA ≤35 g/L and 18 out of 310 patients (6%) in those with SA >35 g/L (*P* < .001). A Cox regression analysis confirmed that SA ≤35 g/L independently predicted PVT (HR: 2.076; 95% CI: 1.140–3.783; *P* = .017), along with platelet count (HR: 0.986; 95% CI: 0.979–0.993; *P* < .001), after adjusting for sex and Child–Pugh classes.

## Discussion

The study provides the first evidence that in cirrhosis hypoalbuminemia, as defined by SA ≤35 g/L, is associated with incident PVT during a follow-up of 2 years.

In the entire cohort, SA values ≤ 35 g/L were detected in 52% of patients. Among the clinical variables examined, the severity of liver disease and the coexistence of liver cancer were associated with SA ≤35 g/L; among the laboratory variables SA values ≤ 35g/L were associated with elevated hs-CRP. This last finding suggests that, in addition to lowered albumin biosynthesis, hypoalbuminemia is also due to the coexistence of chronic inflammation that may oxidize albumin so reducing its blood concentration and impairing its antioxidant property.^11^

As previously reported by the present cohort,^9^ liver cirrhosis is associated with enhanced risk of incident PVT with a rate that is closely related to a history of PVT; thus, the incidence rate of PVT was 6.05 per 100 patient-years in all patients, 4.1 per 100 patient-years in those PVT-free at baseline and 18.9 per 100 patient-years in those with PVT at admission; other clinical and laboratory variables associated with PVT were HCC and thrombocytopenia.^9^ Here we show that in the same cohort SA values ≤ 35 g/L at admission were independently associated with incident PVT during the follow-up; of note, this association persisted even if patients with previous PVT were excluded from the analysis reinforcing the hypothesis that patients with SA ≤35 g/L are more prone to experience PVT.

The biological plausibility of the association between SA ≤35 g/L and PVT relies on the fact that albumin possesses antiplatelet properties via a mechanism related to its antioxidant effect; thus, albumin downregulates Nox2, the most important cellular enzymatic pathway eliciting the production of ROS and the formation of pro-aggregating eicosanoids such as thromboxane A_2_ and F2-isoprostanes.^20^ Accordingly, preliminary short-term albumin infusion in cirrhotic patients resulted in Nox2 downregulation, ROS lowering and platelet aggregation inhibition^12^; further study with larger samples size is needed to confirm this finding. Also, albumin encompasses anticoagulant activity by inhibiting fibrin polymerization, potentiating antithrombin III activity, and modulating factor V, factor VIII, and fibrinogen by liver cells.^21^^,^^22^ Studies in human showed that hypoalbuminemia is associated with a hypercoagulable state as depicted by high levels of D-dimer, a marker of systemic fibrinolysis; thus, in patients at risk of thrombosis such as those with Covid-19 and those with acutely ill medical problems at risk of venous and arterial thrombosis hypoalbuminemia inversely correlated with D-dimer and significantly associated with incident venous and arterial thrombotic events.^23^^,^^24^ A further support to the anticoagulant property of albumin was provided by a pilot study in Covid-19 patients supplemented with albumin showing a significant reduction of D-dimer coincidentally with an increase of SA reinforcing the hypothesis that albumin exerts an anticoagulant activity in vivo.^25^ D-dimer is also elevated in cirrhotic patients and significantly correlates with thrombin generation suggesting that it may be secondary to an ongoing prothrombotic state.^1^^,^^4^ Hence, the inverse association between SA ≤35 g/L and D-dimer observed in our cohort could be interpreted as hypoalbuminemia-related hypercoagulable state, even if heightened D-dimer in cirrhosis may be partly dependent upon a concomitant systemic proteolysis.^1^

Together with SA ≤35 g/L, incident PVT was also correlated with low platelet counts and male sex. Thrombocytopenia in cirrhotic patients is indicative of advanced liver failure; nevertheless, even when present in reduced numbers, platelets seem to exhibit heightened activation and may contribute to the hypercoagulative state that may favor PVT.^2^

The finding of an association between male sex and PVT is intriguing. However, this observation could be influenced by the composition of our cohort, where males were more likely to be afflicted by HCC, a condition known to be linked with PVT.^19^ Importantly, in patients without HCC, male sex did not predict a higher incidence of PVT. Thus, the relationship between sex and PVT warrants exploration in additional studies with larger sample sizes.

The study has implications and limitations. The fact that albumin possess anticoagulant property and that previous and present studies found an inverse correlation with D-dimer gives novel insights on the mechanism accounting for the hypercoagulable state of liver cirrhosis; thus, hypoalbuminemia could reduce the anticoagulant property of the blood, favor clotting and platelet activation and eventually precipitate thrombotic events. The present study suggests that patients with SA ≤35 g/L could be regarded at potential risk of future PVT and, thereby, candidates to appropriate anticoagulant prophylaxis such as low molecular weight heparin, that, given at prophylactic dosage, has been shown to lower incident PVT in PVT-free enrolled cirrhotic patients during a follow-up of 1 year^26^; however, considering the retrospective nature of the present study prospective study with larger sample size is needed to support this hypothesis. Albumin supplementation may be another interesting option to counteract the hypercoagulable state and lower the thrombotic risk in cirrhotic patients with hypoalbuminemia. Previous studies reported nonunivocal effects of albumin supplementation on survival of cirrhotic patients^26^; further study could be done to see if albumin supplementation is also able to prevent PVT in at risk patients.

Limitations of the present study include that D-dimer and hs-CRP were exclusively analyzed within specific sub-groups of patients. Moreover, this study was not designed to assess anticoagulation therapy; instead, its administration was at the discretion of the treating physician at each center. As a result, our dataset encompasses solely baseline information on anticoagulants, lacking comprehensive details regarding their doses, their continuous usage or discontinuation throughout the follow-up period. The anticoagulation treatment in the context of PVT in cirrhotic patients is still a matter of debate and warrants further investigation.^27^^,^^28^ Additionally, we lacked data regarding hospitalization or treatment received during the follow-up period. Furthermore, due to the relatively small sample size, both the initial occurrence of PVT and the recurrence of PVT were considered as events during the follow-up in the primary analysis. However, acknowledging our prior demonstration that PVT recurrence is more prevalent than new incident PVT,^9^ we conducted a supplementary analysis excluding patients with PVT at baseline. Despite the reduced number of events, this analysis confirmed the association between SA ≤35 g/L and the risk of new PVT. It is worth noting that only a substantially larger cohort of patients and randomized controlled trials employing anticoagulant agents or albumin supplementation could provide more definitive insights into the intricate relationship between low SA, factors that influence its serum levels, the risk of new incident PVT, and the effectiveness of anticoagulant treatments. Finally, the study has been done in an Italian population; thereby our findings must be confirmed by another population of different country.

## Conclusion

In cirrhosis the relationship between hypoalbuminemia and thrombosis provides novel insight on the mechanism accounting for hypercoagulable state of cirrhotic patients; hypoalbuminemia could predispose to hypercoagulable state so favoring thrombosis precipitation in the portal circulation. Patients with SA ≤35 g/L are at higher risk of PVT and should be regarded as potential candidates for anticoagulant prophylaxis.

## Supplementary Materials

Online Appendix

Tables A1 and A2
