# Supplementary material for: Hypoalbuminemia and Risk of Portal Vein Thrombosis in Cirrhosis
Source: Gastro Hep Adv. 2024 Mar 13;3(5):646–53. doi: 10.1016/j.gastha.2024.03.006 (PMC11330931; doi:10.1016/j.gastha.2024.03.006)
Supplement: Online Appendix [file mmc1.docx]

**ONLINE APPENDIX**

Palasciano Giuseppe, D’Alitto Felicia, Palmieri Vincenzo Ostilio, Santovito Daniela (Clinica Medica, UOC Medicina Interna Universitaria ‘‘A. Murri’’), Di Michele Dario, Croce Giuseppe (U.O.C. di Medicina Interna - Ospedale ‘‘G. Mazzini’’ ASL Teramo), Sacerdoti David, Brocco Silvia, Fasolato Silvano, Cecchetto Lara, Bombonato Giancarlo (Department of Medicine, University of Padova, Padova, Italy), Bertoni Michele, Restuccia Tea (U.O.C. Medicina Interna 2, Nuovo Ospedale di Prato), Andreozzi Paola, Liguori Maria Livia (Centro di Medicina Predittiva, prevenzione e cura della malattie cronico degenerative e controllo dei fattori di rischio, Azienda Policlinico Umberto I, Roma), Perticone Francesco, Caroleo Benedetto, Perticone Maria, Staltari Orietta (Department of Medical and Surgical Sciences, University of Catanzaro; UO Malattie Cardiovascolari Geriatriche, Policlinico Mater Domini di Catanzaro; Dipartimento di Medicina Sperimentale e Clinica, Università Magna Graecia di Catanzaro; Dipartimento Scienze della Salute, Università Magna Graecia di Catanzaro), Manfredini Roberto, De Giorgi Alfredo (UOC Clinica Medica, Azienda Ospedaliero - Universitaria di Ferrara), Averna Maurizio, Giammanco Antonina (Dipartimento Biomedico di Medicina Interna e Specialistica, Università degli Studi di Palermo), Granito Alessandro, Pettinari Irene, Marinelli Sara, Bolondi Luigi (U.O. Medicina Interna, Dipartimento di Scienze Mediche e Chirurgiche, Università di Bologna), Falsetti Lorenzo, Salvi Aldo (Medicina Interna Generale e Subintensiva - Ospedali Riuniti di Ancona), Durante-Mangoni Emanuele, Cesaro Flavio, Farinaro Vincenza, Ragone Enrico (Medicina Infettivologica e dei Trapianti, Seconda Università di Napoli), Morana Ignazio (U.O. Medicina Interna Area Critica, ARNAS ‘‘Garibaldi’’ Catania), Andriulli Angelo, Ippolito Antonio, Iacobellis Angelo, Niro Grazia, Merla Antonio (Gastroenterologia ed Endoscopia Digestiva-Casa Sollievo della Sofferenza –Ospedale- IRCCS San Giovanni Rotondo), Raimondo Giovanni, Maimone Sergio, Cacciola Irene, Varvara Doriana (Division of Clinical and Molecular Hepatology, Department of Internal Medicine University Hospital of Messina), Drenaggi Davide, Staffolani Silvia (Clinica Malattie Infettive Tropicali, Parassitologia, Epatiti croniche Azienda Ospedaliero Universitaria ‘‘Ospedali Riuniti’’), Picardi Antonio, Vespasiani-Gentilucci Umberto, Galati Giovanni, Gallo Paolo (UOS Medicina Clinica, Epatologia Università Campus Bio-Medico), Davì Giovanni, Schiavone Cosima, Santilli Francesca, Tana Claudio (UO Patologia Medica, Ospedale Clinicizzato ‘‘SS. Annunziata’’ Chieti), Licata Anna, Soresi Maurizio (UOC di Medicina Interna ed Epatologia, DIBIMIS, Università di Palermo), Bianchi Giovanni Battista, Carderi Isabella (UOC di Medicina Generale dell’Ospedale ‘‘SS. Capitanio e Gerosa’’ di Lovere), Pinto Antonio, Tuttolomondo Antonino (Unità Operativa Complessa di Medicina Interna e Cardioangiologia, Dipartimento Biomedico di Medicina Interna e Specialistica, Università degli Studi di Palermo), Ferrari Giovanni (U.O.C. di Medicina Interna, Ospedale ‘‘SS.Annunziata’’ di Varzi, A.O della Provincia di Pavia), Gresele Paolo, Fierro Tiziana, Morelli Olivia (S.S. di Diagnosi e Terapia delle Malattie Emorragiche e Laboratorio delle Malattie Tromboemboliche; S.C. Medicina Interna e Cardiovascolare, Dipartimento di Medicina; S.C. Gastroenterologia ed Epatologia, Università - Azienda Ospedaliera di Perugia), Laffi Giacomo, Romanelli Roberto Giulio, Arena Umberto, Stasi Cristina (Azienda Ospedaliero Universitaria Careggi , Unità Operativa Complessa Medicina Interna ed Epatologia, Firenze), Gasbarrini Antonio, Gargovich Matteo, Zocco Maria Assunta, Riccardi Laura, Ainora Maria Elena (U.O.C. Medicina Interna e Gastroenterologia, Policlinico Gemelli, Roma), Capeci William, Martino Giuseppe Pio, Nobili Lorenzo (Clinica Medica Generale - AOU ‘‘Ospedali Riuniti’’ Ancona), Cavallo Maurizio (Medicina di Arzignano/Lonigo –ULSS 5 Ovest Vicentino), Frugiuele Pierluigi, Greco Antonio (Struttura Complessa di Medicina Interna e Reumatologia Azienda Ospedaliera di Cosenza), Pietrangelo Antonello, Ventura Paolo, Cuoghi Chiara, Marcacci Matteo (Divisione Medicina Interna 2 / CEMEF e Centro Dipartimentale di Ricerca Epatologica Avanzata ‘‘Mario Coppo’’, Azienda Ospedaliero-Universitaria Policlinico di Modena), Serviddio Gaetano, Vendemiale Gianluigi, Villani Rosanna, Gargano Ruggiero (Centro Universitario per la ricerca e cura delle malattie epatiche - Clinica Medica Universitaria, Foggia), Vidili Gianpaolo, Di Cesare Valentina, Masala Maristella, Delitala Giuseppe (Clinica Medica , Azienda Ospedaliero Universitaria Sassari), Invernizzi Pietro (Center for Autoimmune Liver Diseases, Humanitas Clinical and Research Center, Rozzano), Di Minno Giovanni, Tufano Antonella (Centro di Coordinamento Regionale per le Emocoagulopatie, AOU Federico II Napoli), Purrello Francesco, Privitera Graziella (Dipartimento di Medicina Clinica e Sperimentale Università di Catania, UOC di Medicina Interna Ospedale Garibaldi-Nesima, Catania), Forgione Alessandra, Curigliano Valentina (IRCCS San Raffaele Pisana Roma), Senzolo Marco, Rodríguez-Castro Kryssia Isabel (Unità Trapianto Multiviscerale, Gastroenterologia, Università Ospedale Di Padova), Giannelli Gianluigi (Dipartimenti delle Emergenze e dei Trapianti di Organo, Sezione di Medicina Interna, Allergologia ed Immunologia Clinica, Bari), Serra Carla (Ecografia diagnostica ed interventistica, Dipartimento delle Insufficienze d’organo e dei Trapianti, Policlinico S.Orsola-Malpighi, Bologna), Neri Sergio (UOC di Medicina Interna ‘‘A. Francaviglia’’, Policlinico Universitario di Catania), Pignataro Pietro (UOC I Medicina Interna, AOU Policlinico Vittorio Emanuele, Catania), Rizzetto Mario, Debernardi Venon Wilma (UOADU Gastroepatologia, Azienda Ospedaliera San Giovanni Battista Di Torino), Svegliati Baroni Gianluca (Clinica di Gastroenterologia, Ancona), Bergamaschi Gaetano, Masotti Michela, Costanzo Filippo, Corazza Gino Roberto (Fondazione IRCCS Policlinico San Matteo, University of Pavia, Italy), Caldwell Stephen Hugh (Division of Gastroenterology and Hepatology, School of Medicine, University of Virginia, Charlottesville, USA), Angelico Francesco, Del Ben Maria, Napoleone Laura, Polimeni Licia, Proietti Marco, Raparelli Valeria, Romiti Giulio Francesco, Ruscio Eleonora, Severoni Andrea, Talerico Giovanni, Toriello Filippo, Vestri Annarita (Sapienza University of Rome, Italy).
