# Supplementary material for: Hypoalbuminemia and Risk of Portal Vein Thrombosis in Cirrhosis
Source: Gastro Hep Adv. 2024 Mar 13;3(5):646–53. doi: 10.1016/j.gastha.2024.03.006 (PMC11330931; doi:10.1016/j.gastha.2024.03.006)
Supplement: Tables A1 and A2 [file mmc2.docx]

**Supplemental Table 1. Baseline clinical characteristics of patients with available high-sensitivity C-Reactive Protein (hs-CRP) data.**

| Variables | N=264 |
| --- | --- |
| Age, years | 64.9 ±12.0 |
| Male sex, n (%) | 183 (69) |
| Etiology, n (%) |  |
| - Alcohol, n (%) | 85 (32) |
| - Viral, n (%) | 104 (39) |
| - Autoimmune, n (%) | 8 (3) |
| - NASH/metabolic, n (%) | 9 (3) |
| - Mixed, n (%) | 34 (13) |
| - Others/unknown, n (%) | 24 (9) |
| Child–Pugh class |  |
| Class A, n (%) | 97 (37) |
| Class B, n (%) | 113 (43) |
| Class C, n (%) | 54 (21) |
| MELD score | 12 [9-15] |
| HCC, n (%) | 72 (27) |
| Bilirubin, (mg/dL) | 1.5 [0.9-2.8] |
| PT-INR | 1.35 ± 0.35 |
| Serum creatinine (mg/dL) | 0.8 [0.7-1.1] |
| Platelet count (×10^3^/µL) | 97 [69-147] |
| Ascites |  |
| - Absent, n (%) | 113 (43) |
| - Responsive to diuretic therapy, n (%) | 104 (39) |
| - Refractory, n (%) | 47 (18) |
| Encephalopathy |  |
| - Absent, n (%) | 202 (76) |
| - Mild, n (%) | 52 (20) |
| - Moderate to severe, n (%) | 10 (4) |

MELD: Model for End-Stage Liver Disease; NASH: non-alcoholic steatohepatitis; PT-INR: prothrombin time-international normalized ratio; HCC: Hepatocellular carcinoma. Data are expressed as mean ± standard deviation, or median [interquartile range] or number (percentage).

**Supplemental Table 2. Baseline clinical characteristics of patients with available D-dimer data.**

| Variables | N=112 |
| --- | --- |
| Age, years | 64.0 ± 11.7 |
| Male sex, n (%) | 76 (68) |
| Etiology, n (%) |  |
| - Alcohol, n (%) | 28 (25) |
| - Viral, n (%) | 54 (48) |
| - Autoimmune, n (%) | 6 (5) |
| - NASH/metabolic, n (%) | 5 (4) |
| - Mixed, n (%) | 15(13) |
| - Others/unknown, n (%) | 4 (4) |
| Child–Pugh class |  |
| Class A, n (%) | 57 (51) |
| Class B, n (%) | 42 (37) |
| Class C, n (%) | 13 (12) |
| MELD score | 11 [9-14] |
| HCC, n (%) | 24 (21) |
| Bilirubin, (mg/dL) | 1.4 [0.8-2.3] |
| PT-INR | 1.36 ± 0.36 |
| Serum creatinine (mg/dL) | 0.8 [0.7-1.1] |
| Platelet count (×10^3^/µL) | 85 [66-136] |
| Ascites |  |
| - Absent, n (%) | 64 (57) |
| - Responsive to diuretic therapy, n (%) | 40 (36) |
| - Refractory, n (%) | 8 (7) |
| Encephalopathy |  |
| - Absent, n (%) | 94 (84) |
| - Mild, n (%) | 17 (15) |
| - Moderate to severe, n (%) | 1 (1) |

MELD: Model for End-Stage Liver Disease; NASH: non-alcoholic steatohepatitis; PT-INR: prothrombin time-international normalized ratio; HCC: Hepatocellular carcinoma. Data are expressed as mean ± standard deviation, or median [interquartile range] or number (percentage).
